# The Clinical Society of Maryland

**Published:** 1893-02

**Authors:** W. T. Watson

**Affiliations:** Secretary; 1519 N. Broadway; Baltimore


*SOCIETY REPORTS.*

### THE CLINICAL SOCIETY OF MARYLAND.

BALTIMORE, December 2, 1892.

DR. J. E. MICHAEL read a paper entitled

### SYMPHYSIOTOMY—A SUCCESSFUL CASE—A SUGGESTION.

The ancient history of the operation was briefly referred to. Dr. Harris' paper, read before the last meeting of the American Gynecological Society and published in the *American Journal of Obstetrics* in October, 1892, leaves little to be said as to the modern history of the operation. Dr. Harris' table, showing forty-four operations from January, 1886, to July, 1892, by various operators, with one maternal death, three still-born children dying respectively at twelve and seventy-two hours, made a profound impression on the American profession. Dr. Charles Jewett, of Brooklyn, was the first American operator. He operated on September 30, 1892. The child died in twenty-four hours from the effect of long continued pressure. The recovery of the mother was uneventful. Professor Hirst, of Philadelphia, operated October 2, 1892. Child and mother both well. Professor Broomall operated October 7, 1892. Mother and child saved. Dr. Michael operated at the Free Lying-in Hospital of the University of Maryland, October 24, 1892. The patient was a rachitic negress, four feet, six inches high, seventeen years old. Labor began on the morning of the 23d. Dr. Michael saw the patient at 9 P. M. Os barely admitted two fingers. Head large and no sign of engagement. Fœtal and maternal circulation good and general condition of patient satisfactory. It was concluded to wait for greater dilatation and operation was postponed till morning. Operation at 9 : 30 A. M., chloroform anæsthesia. Os still small ; most of the amniotic fluid had escaped and the fœtus was suffering from pressure. Fœtal head obviously large and no possibility of engagement. The bladder was evacuated and then the os uteri dilated until four fingers would enter. The soft tissues were incised down to the symphysis and the attachments of the recti were separated for half an inch on each side. The finger was passed down behind the symphysis until it projected below. The soft parts from the outside below were incised down to the finger tips. An ordinary curved probe-pointed bistoury was passed behind the joint and the cartilage severed. Delivery by Simpson's modification of Tarnier's forceps. Pubic separation at its highest point was two and seven-eighths inches. Notwithstanding all precautions the cervix was lacerated into the vaginal vault, the perineum to the verge of the anus and the anterior vaginal vault into the operation wound. The lacerations repaired at once with catgut. The wound of the symphisis was sewed with gut, the deeper stitches including the pubic ligaments. The surface was powdered with aristol and dressed with iodoform gauze. Broad adhesive strips encircled the pelvis and were covered by a firmly applied bandage. The puerperium was uneventful. On the ninth day the patient was allowed to sit up in bed ; on the eleventh day sat up a little while in a chair ; on the twelfth day could walk well and firmly. At the present time she walks all over the hospital and as firmly as before the operation. The child died on the third day, the death being due to pressure which had occurred previously to the delivery. Dr. Michael, since the operation, has procured a Galbiati knife which he believes would have been of great service in the operation.

Dr. Michael believes that symphysiotomy will not only to a large extent take the place of Cæsarean section and of craniotomy on the living child, in cases of contracted pelvis, but will be of service in the delivery of living children in cases of bad presentation where formerly craniotomy has been resorted to. He has examined this matter experimentally with a fœtus of large size and a pelvis, with the soft parts attached, of comparatively small size. Placing the fœtal head into the pelvis and producing a posterior rotation of the chin, delivery was attempted by forceps, but was found to be utterly impossible. Symphysiotomy was performed, and after the pubic bones were separated, one and one-half inches, the head was easily flexed on the trunk, the occiput brought under the pubic arch and delivery by extension occurred in the usual way.

Dr. Michael was so impressed with the feasibility of this operation that he intends to perform it on the first case of malposition of the head which presents itself to him. In cases where the occiput is posterior and the delivery of the child with forceps is accompanied with a great amount of violence, he thinks that this operation may be indicated. The number of operations which have been performed the present year the world over as collected by Dr. Harris, of Philadelphia, is twenty-six. In this list there is no death of the mother. The statistics are remarkable, both as to the safety of the woman and the healing of the wound.

Dr. Hunter Robb asked Dr. Michael if in his case he had been able to suture the pubic ligaments as described by Leopold, of Dresden, in a case of symphysiotomy.

Dr. Michael said that the ligaments of the pubes offered a very considerable amount of tissue which might be caught with sutures. It would be unwise to depend on sutures, however they were passed. The pressure from the sides as produced by adhesive plasters and a well applied bandage over them is so complete that you get a support which no suture of any kind could supply, and it would not make a very great amount of difference if the ligatures were not applied at all.

DR. ROBB congratulated Dr. Michael upon the success of this case. He thought that symphysiotomy would undoubtedly have a prominent position in obstetric surgery. On account of the simplicity of the operation there will be great danger of its being performed more often than necessary.

The pelvic measurements should be made as carefully as possible with consultants of sufficient experience before operation, just as is done when Cæsarean section is thought of. In some cases the operation is undoubtedly so clearly indicated that immediate action is justifiable, but these cases, he believed, form the large minority. Symphysiotomy does not provide for as many abnormal conditions as Cæsarean section ; for example, where one has to deal with cancerous growths of the cervix, pelvic exostoses, tumors of the uterus, and some deformities of the pelvic bones, it would be useless to do symphysiotomy.

He believed, however, that the operation would perhaps save the lives of many children ; on the other hand, it may leave undesirable results in the mother.

DR. WILLIAM S. GARDNER said that the profession was indebted to Dr. Michael for bringing this subject before them. This operation will almost entirely take the place of craniotomy on the child where the condition is that of contracted pelvis. Of course nobody would dream of doing symphysiotomy for a cancerous cervix or where the obstruction was due to another condition in the pelvis than that of contraction. The operation will also cut in very largely upon the Cæsarean section, especially those Cæsarean sections done in the United States. The fact is very well known that we have in the United States a very small number of extremely contracted pelves and that a large percentage of the Cæsarean sections that have been done were upon women who had only what is known as the "relative indication." With reference to the suturing of the pubic bones, Leopold remarked at the time he was stitching the wounds that he did not consider the stitching of very much value and that he placed his main reliance upon the external bandage. The bandage which he used was made of heavy ducking and was fastened by a buckle resembling an ordinary suspender buckle. The hips were paded with towels and this bandage was drawn over them very tightly, in fact so tightly that it produced sores on both the crests of the ilium in his first case. In Germany and in most of the European countries they have a large number of cases where the degree of contraction is so extreme that symphysiotomy is not a practical operation. The Galbiati knife is probably of great convenience to the operator.

DR. NORMENT was particularly interested in the suggestion which Dr. Michael made as to the performance of symphysiotomy to save the necessity of craniotomy upon the living child on account of malposition of the fœtus. He had once been compelled to do craniotomy in a face presentation, chin posterior. He saw very readily wherein the operation of symphysiotomy would give relief to that condition. It is an operation which is not to be attempted by any and every one. As Dr. Robb has said it should not be resorted to until after the judgment of able consultation has been passed upon it. The necessity of getting consultation or of getting some one else to do the operation would take away from it a great part of its service, because often in private practice the delay necessary to secure these things would be dangerous to the mother.

Dr. Branham thanked Dr. Michael for bringing up the subject. He thought that the operation was still on trial and it would not be best to jump to the conclusion, as is so often done when these operations are revived or first brought out, that it is to be a continued success. The statistics on the subject as presented are extremely favorable to the operation and it seems undoubtedly that the very recent operations have been quite successful, but even since the introduction of antiseptic methods the operations done between the years 1880 and 1886 and collected by Morrisani give a mortality of 8 in 18 operations. Of course the mortality seems to have been reduced almost to nothing, but I am inclined to think that more than likely the favorable cases have been reported and the unfavorable cases have not. It hardly seems probable that an operation could be so much improved since 1886. A good many cases which have gotten well have been followed by chronic diseases of the bones about the pelvis. The probabilities are that better antiseptic precautions have diminished the frequency of this sequel. It is more than probable that there will be a certain number of cases in which more or less permanent injury will result. The operation will doubtless have a future, still we should not conclude, until these cases have been done for a long time and the final results as to the conditions of the pelvic bones are known, that this operation is going to take the place of Cæsarean section. As far as operation in cases of impaction is concerned, if it can be done in time to save the child it is a very good thing and will doubtless be carried out in a great many cases.

DR. MICHAEL: Of course the operation of symphysiotomy in tumor and cancer and that sort of thing is not to be thought of. It only applies to such conditions as can be changed by the effect upon the bones. The discussion of the question of symphysiotomy in a case of malposition can only come up when the head is down and impacted. With a child dead and posterior chin or a child nearly dead, of course symphysiotomy is not to be thought of; but with a jammed head and a living child where the alternative rests between craniotomy and symphysiotomy the latter is to be elected. Dr. Branham's position in regard to conservatism is a proper one. We should always receive new operations with a certain amount of scepticism and it is very well to look closely into the results of operations before jumping to conclusions. What he says in regard to operations reported between 1880 and 1886 is perfectly correct, but if he will look over hernia or any other class of operations in which much tissue is involved or cavities of importance invaded, which were made within the same period, he will find that the mortality was greater than it is to-day. Even the operation of Cæsarean section has improved wonderfully since the period mentioned. As to the matter of reporting only favorable cases, we certainly have a complete record of the work of men who are prominent in these branches and in whom the suppression of unsuccessful cases would be simply disgraceful. I am firmly convinced from Dr. Harris' figures that there is an amount of improvement in the results of symphysiotomy due to antisepsis that is represented by the reported cases. Of course there must be concealed cases of any operation of gravity, but the reports we have here are fully as reliable as reports of any subject of this sort. We have not here an operation which is on trial. When we can present a record of fifty-two cases it strikes me that the utility of the operation for saving life has been demonstrated. If we do not accept this number of cases as proving it, then I do not see how we are ever going to prove it. We should not hinder the wheels of progress by referring to the bad work of ignorant people. I think the utility of the operation is demonstrated.

DR. HUNTER ROBB read a paper on

HYSTEROMYOMECTOMY FOR LARGE MYOMATA OF THE UTERUS.

Dr. Robb strongly advocated the intra-peritoneal method of treating the pedicle after the removal of the uterus. The dangers of sepsis and hemorrhage with improved technique are less than when the extra-peritoneal method is employed and are not much greater than in an ordinary ovariotomy. The various devices for controlling hemorrhages were considered. The danger from sepsis from the cervical canal can most surely be obviated by curetting both the uterine and cervical mucosa several days prior to the removal of the tumor. At the same time the cavity of the uterus can be cauterized gently with the small point of a Paquelin cautery and a strip of 10 per cent. iodoform gauze packed in to be removed the day before the operation. The vagina can be made sterile by irrigation twice daily for two or three days prior to the operation with $\frac{1}{3}$ per cent. warm solution of carbolic acid. The vagina between douches should be packed with iodoform gauze. The external genitals should be rendered aseptic. After removal of the tumor, the cervical canal should be sterilized by plunging the Paquelin cautery well into the lumen of the canal. The results obtained by this method in the Johns Hopkins Hospital within the past year and a half have been more satisfactory than where other methods were employed. The period of convalescence is shortened and there is not nearly so much danger of the hernial complications that are apt to follow other methods. Dr. Robb now drops the pedicle in every case of hysteromyomectomy. The paper was illustrated by large bromide prints showing the different steps taken in the operation described.

DR. W. P. CHUNN thought that a method which had not been mentioned by Dr. Robb, namely, where no pedicle was left at all, was a particularly good variety and one which would sometime come into a great deal of use. He believed that wherever it was possible to get rid of the extra-peritoneal method it should be done for with the greatest care we are apt to have some sepsis. In cases where hemorrhage is feared he advised that an extra suture be passed through the stump and be allowed to come out at the lower end of the abdominal wound so that it can be gotten at more readily if hemorrhage occurs.

DR. W. S. GARDNER described a method of dealing with the stump by covering it with flaps of peritoneum. He advised cutting out a rim of tissue around the cervical canal, after the use of the Paquelin cautery, so as to get fresh surfaces instead of the cauterized. Where there is considerable mass of tissue in the stump it is difficult to tie it tight enough with ligatures to control hemorrhage. The uterine tissue under pressure will decrease in size and the ligatures become loose. A useful measure to prevent hemorrhage is to pass a stitch around each uterine artery.

DR. ROBB : In my paper I have only considered whether we should drop or in some way fix the pedicle, and I have endeavored to show, that with our improved technique we are able to drop the pedicle and not treat it by any form of fixation in the abdominal wound. The entire removal of the uterus practically leaves no pedicle, and therefore I have not mentioned this method, nor the method of vaginal fixation described by Dr. Byford, of Chicago.

The point of greatest interest to abdominal surgeons is whether the pedicle is to be treated extra-peritoneally or intra-peritoneally. The dropping of the pedicle is the ideal procedure and does much to simplify the operation ; I think it is generally conceded that our technique must be such that this method can be employed.

The uterine arteries can be ligatured in some cases in the way which Dr. Gardner mentions.

DR. EDWIN K. BALLARD exhibited a large uterine fibroid removed by saw-spoon. The patient, Mrs. B., aged 53 years, entered the Hospital for Women of Maryland, suffering from a sub-mucous fibroid about the size of a foetal head. Ergot was given for a number of weeks and the os dilated under this treatment to the size of a silver dollar. The patient had formerly suffered from profuse hemorrhages and was weak and anæmic. On November 29th, Dr. H. P. C. Wilson, assisted by

Drs. R. T. Wilson, E. K. Ballard and W. McL. Yost, operated for the removal of the growth. The pedicle was broad, sessile and attached mainly to the fundus and posterior wall of the uterus. It was found utterly impossible to use the ecraseur. The Thomas saw-spoon was passed the full length of the shank, eight inches, but failed to reach the base of the tumor. Nothing remained but to cut away portions of the protruding mass and remove it piecemeal. Accordingly an attack was made on the main body of the growth from which a piece was sawn loose, pulled out and cut off with saw-scissors. The next presenting portion was seized by vulsella, dragged down and detached in a similar manner. After a number of sections had been thus removed the remaining pedicle was drawn down into view and acted upon by the saw-scoop with which it was after much difficulty, on account of the density, cut away. By this time the patient had lost a very large quantity of blood and was profoundly collapsed but by vigorous stimulation with amyl-nitrite by inhalation, together with whisky and digitalis hypodermically, she was somewhat revived. Cervix and perineum were both torn during the operation. After removal of growth, the uterus was flushed with hot water and mopped with a mixture of Monsel's solution, carbolic acid and glycerine as an antiseptic and styptic. The patient was carefully put to bed and stimulation continued. She promptly rallied and has progressed favorably.

W. T. WATSON, Secretary.

1519 *N. Broadway.*

